# GSR is not essential for the maintenance of antioxidant defenses in mouse cochlea: Possible role of the thioredoxin system as a functional backup for GSR

**DOI:** 10.1371/journal.pone.0180817

**Published:** 2017-07-07

**Authors:** Chul Han, Mi-Jung Kim, Dalian Ding, Hyo-Jin Park, Karessa White, Logan Walker, Tongjun Gu, Masaru Tanokura, Tatsuya Yamasoba, Paul Linser, Richard Salvi, Shinichi Someya

**Affiliations:** 1 Department of Aging and Geriatric Research, University of Florida, Gainesville, FL, United States of America; 2 Center for Hearing and Deafness, State University of New York at Buffalo, NY, United States of America; 3 Whitney Laboratory, University of Florida, St Augustine, FL, United States of America; 4 Bioinformatics, Interdisciplinary Center for Biotechnology Research, University of Florida, Gainesville, FL, United States of America; 5 Department of Applied Biological Chemistry, University of Tokyo, Yayoi, Tokyo, Japan; 6 Department of Otolaryngology, University of Tokyo, Hongo, Tokyo, Japan; Universidade de Sao Paulo Instituto de Biociencias, BRAZIL

## Abstract

Glutathione reductase (GSR), a key member of the glutathione antioxidant defense system, converts oxidized glutathione (GSSG) to reduced glutathione (GSH) and maintains the intracellular glutathione redox state to protect the cells from oxidative damage. Previous reports have shown that *Gsr* deficiency results in defects in host defense against bacterial infection, while diquat induces renal injury in *Gsr* hypomorphic mice. In flies, overexpression of *GSR* extended lifespan under hyperoxia. In the current study, we investigated the roles of GSR in cochlear antioxidant defense using *Gsr* homozygous knockout mice that were backcrossed onto the CBA/CaJ mouse strain, a normal-hearing strain that does not carry a specific *Cdh23* mutation that causes progressive hair cell degeneration and early onset of hearing loss. *Gsr*^-/-^ mice displayed a significant decrease in GSR activity and GSH/GSSG ratios in the cytosol of the inner ears. However, *Gsr* deficiency did not affect ABR (auditory brainstem response) hearing thresholds, wave I amplitudes or wave I latencies in young mice. No histological abnormalities were observed in the cochlea of *Gsr*^-/-^ mice. Furthermore, there were no differences in the activities of cytosolic glutathione-related enzymes, including glutathione peroxidase and glutamate-cysteine ligase, or the levels of oxidative damage markers in the inner ears between WT and *Gsr*^-/-^ mice. In contrast, *Gsr* deficiency resulted in increased activities of cytosolic thioredoxin and thioredoxin reductase in the inner ears. Therefore, under normal physiological conditions, GSR is not essential for the maintenance of antioxidant defenses in mouse cochlea. Given that the thioredoxin system is known to reduce GSSG to GSH in multiple species, our findings suggest that the thioredoxin system can support GSSG reduction in the mouse peripheral auditory system.

## Introduction

The antioxidant defenses consist of antioxidant enzymes such as superoxide dismutase (SOD), catalase, glutathione peroxidase (GPX) and peroxiredoxin, and low-molecular weight antioxidants such as glutathione [1]. Glutathione (γ-glutamyl-cysteinyl-glycine) plays important roles in antioxidant defense and protects cells from ROS (reactive oxygen species) [2–4]. Under normal physiological conditions, glutathione is found mostly in the reduced form [5]. The GSH/GSSG redox couple is thought to be an intracellular determinant of the antioxidant capacity because the abundance of GSH (~10–15 mM) is three to four orders of magnitude higher than the other reductants such as NADPH and reduced thioredoxin [4, 6]. GSH can directly scavenge certain free radicals and ROS such as hydroxyl radical, lipid peroxyl radical, hypochlorous acid, peroxynitrite, and hydrogen peroxide [1, 2]. GSH also scavenges ROS through serving as a co-factor for GPX. In such reactions, GSH is oxidized to GSSG, which is then reduced back to GSH by glutathione reductase (GSR) [3, 7, 8]. Furthermore, GSH plays an important role in detoxifying toxic chemicals by serving as a co-factor for glutathione transferase (GST) [9, 10].

GSR is a homodimeric flavoprotein with subunit size of 52.4 kDa [3, 7, 8]. GSR functions as a dimeric disulfide oxidoreductase and uses a FAD (flavin adenine dinucleotide) prosthetic group and NADPH to reduce one molecule of GSSG to two molecules of GSH. Because the holoenzyme consists of apoGSR and FAD, the lack of GSR activity can be caused by inherited mutations in the *GSR* gene or deficiency of FAD or vitamin B_2_ in the diet. In patients suffering from Meniere’s disease whose symptoms include fluctuating hearing loss, a significant decrease in both plasma and lymphocyte GSH/GSSG ratios were observed [11]. Among the antioxidant defense systems existing in cochlear cells, the glutathione system is thought to be the major defense system for the protection of the cochlea [11–17]. Depletion of endogenous GSH by BSO (buthionine sulfoximine), an inhibitor of glutamate-cystein ligase, promotes noise-induced hearing loss (NIHL) in guinea pigs [15]. In chinchilla, the activities of GSR and GCL are elevated in the organ of Corti and stria vascularis of the cochlea following acute noise exposure [16], while *Gpx1*^-/-^ mice exhibit significantly more sensory hair cell loss and greater ABR threshold elevation after noise exposure compared to wild-type control mice [18]. Furthermore, *Gsr* deficiency results in defects in host defense against bacterial infection [19], while the herbicide diquat, known to generate superoxide, induces renal injury in *Gsr* hypomorphic mice [20]. In flies, overexpression of *GSR* extended lifespan under hyperoxia [21]. Together, these reports support the idea that GSR plays critical roles in protecting cochlear cells against ROS. In the current study, we tested the hypothesis that young *Gsr* knockout mice would show evidence of hearing loss and cochlear pathology compared to WT mice. To determine the roles of GSR in the antioxidant defense in the cochlea of mice under normal physiological conditions, *Gsr*^+/-^ mice were backcrossed for 6 generations onto the CBA/CaJ mouse strain, a normal-hearing strain that does not carry a specific *Cdh23* mutation that causes progressive hair cell degeneration and early onset of hearing loss [22]. This backcrossing was needed because the original background of *Gsr* KO mice was the 129SvEvBrd/C57BL/6J strain, and the C57BL/6J mouse strain carries the *Cdh23* mutation and is known to display progressive and early-onset hair cell degenerations and hearing loss [22].

## Materials and methods

### Animals

*Gsr*^+/-^ mice were generated by Lexicon Genetics and obtained from the Mutant Mouse Resource Research Centers (MMRRC) (https://www.mmrrc.org/catalog/sds.php?mmrrc_id=11712). The *Gsr* knockout allele was generated by inserting a gene trap cassette (5174 nucleotides) into the intron 1. RT-PCR analysis revealed that the transcript was absent in the homozygous mutant. The original background of the *Gsr*^+/-^ mice was the 129SvEvBrd /C57BL/6 hybrid strain. Male and female CBA/CaJ mice were obtained from Jackson Laboratory (https://www.jax.org/strain/000654). All animal studies were conducted at the AAALAC-approved Animal Facility in the University of Florida. Experiments were performed in accordance with protocols approved by the University of Florida Institutional Animal Care and Use Committee. Both male and female *Gsr*^+/+^, *Gsr*^+/-^, and *Gsr*^-/-^ littermates were used in the current study.

### Genotyping and DNA sequencing

#### *Gsr* genotyping

*Gsr*^+/-^ males were mated with *Gsr*^+/-^ females, and their offspring were genotyped with DNA extracted from a tail clip obtained at weaning. The following primers were used for genotyping: Gsr-F 5’-AGTCACAAGCTGGGTGGCACTTGC-3’; Gsr-R 5’-ACTCCTCCACGCCAGAACCCTC-3’; Gsr-KO-F 5’-AAATGGCGTTACTTAAGCTAGCTTGC-3’. The PCR cycling parameters were as follows: 94°C for 5 min; 9 cycles of 94°C for 15 s, 65°C for 30 s, 72°C for 40 s; 29 cycles of 94°C for 15 s, 55°C for 30 s, 72°C for 40 s; 72°C for 5 min. PCR products were separated on 1.5% agarose gel and the expected band size for wild type (WT) and knockout allele were 342 and 292 bps, respectively.

#### *Cdh23* genotyping

**Fig 1:**
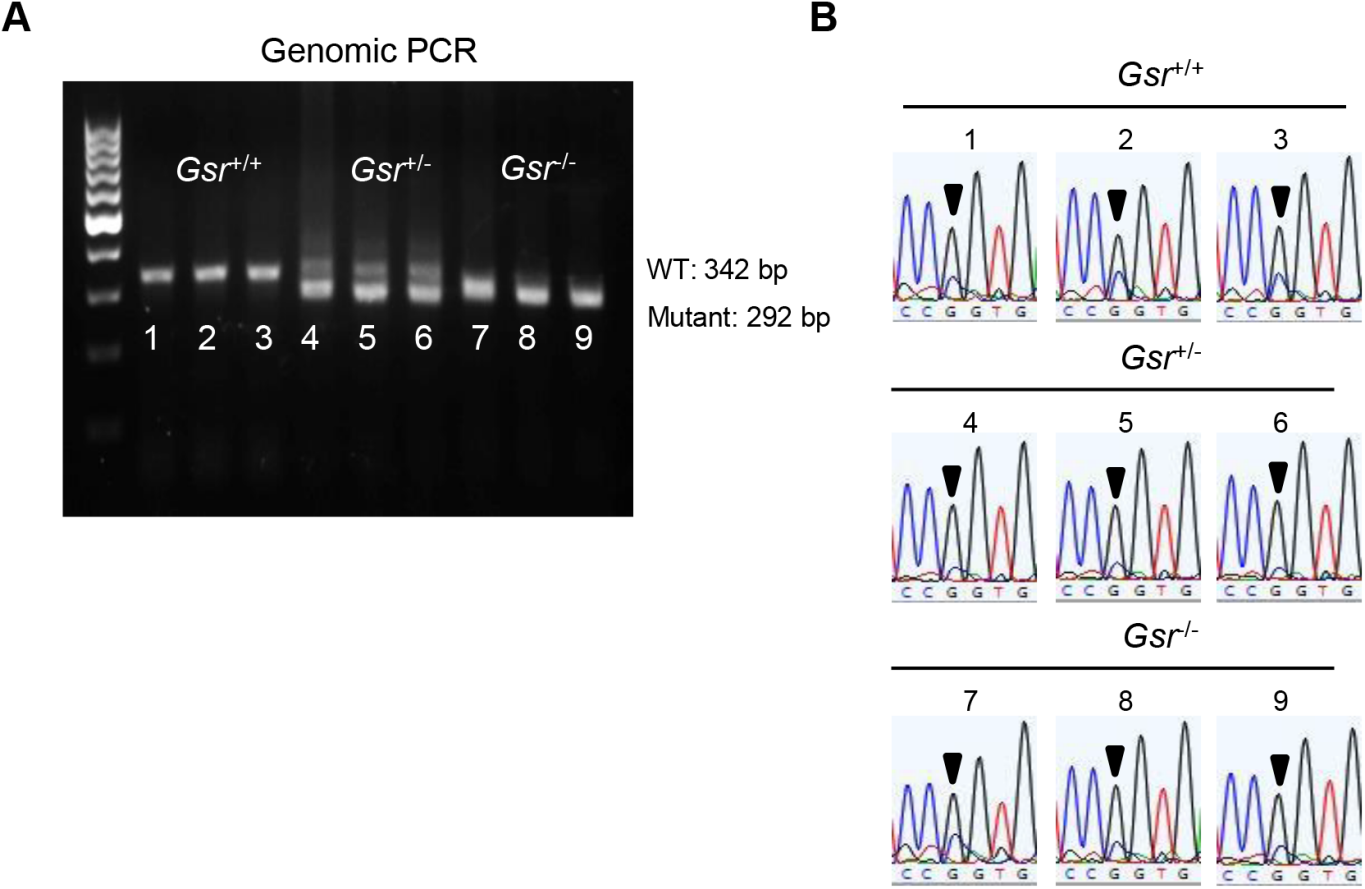
Genotyping of *Gsr*^+/+^, *Gsr*^+/-^, and *Gsr*^-/-^ mice. (A) PCR products were separated on 1.5% agarose gel and the expected band sizes for WT and mutant alleles were 342 and 292 bps, respectively. (B) The *Cdh23* gene in *Gsr*^+/+^, *Gsr*^+/-^, and *Gsr*^-/-^ mice (n = 3 each) was sequenced. All the mice examined had the *Cdh23*^753G/753G^ genotype. Arrows indicate the *Cdh23*^753G^ allele.

Male and female *Gsr*^+/-^ mice have been backcrossed for 6 generations onto the CBA/CaJ mouse strain that does not carry the recessive AHL-susceptibility allele (*Cdh23*^753A^). To confirm that *Gsr*^+/+^, *Gsr*^+/-^, and *Gsr*^-/-^ mice have the same *Cdh23*^753G/753G^ genotype for *Cdh23*, we amplified by PCR and sequenced the region containing the 753rd nucleotide in the *Cdh23* gene (N = 3 each *Gsr*^+/+^, *Gsr*^+/-^, and *Gsr*^-/-^ mice) (Fig 1B). The following primers were used for the PCR reaction: Cdh23-F 5’- GATCAAGACAAGACCAGACCTCTGTC-3’; Cdh23-R 5’-GAGCTACCAGGAACAGCTTGGGCCTG-3’. The size of amplified PCR product was 360 bps.

### Body weight

The body weight of the *Gsr*^+/+^ and *Gsr*^-/-^ mice was measured at 3–5 months of age.

### ABR hearing test

Auditory brainstem responses (ABRs) were measured with a tone burst stimulus at 4, 8, 16, 32, 48 and 64 kHz using an ABR recording system (Tucker Davis Technologies) at 3–5 months of age as previously described [23]. Mice were anesthetized with a mixture of xylazine hydrochloride (10 mg/kg, i.m.) (Phoenix Urology of St. Joseph) and ketamine hydrochloride (40 mg/kg, i.m.) (Phoenix Urology of St. Joseph) and placed on a warm heating pad. Needle electrodes were placed subcutaneously at the vertex (non-inverting or active), ipsilateral ear (reference), and contralateral ear (ground). At each frequency, the sound level was decreased in 10-dB steps from 90 dB SPL to 10 dB SPL (sound pressure level). A hearing threshold was defined as the lowest level that produced a noticeable ABR response. In mice, auditory brainstem responses typically consist of five vertical positive waves (waves I, II, III, IV, and V) [24]. In rodents, wave I represents activities from the auditory nerve, while waves II-V represent neural transmission within the central auditory system. ABR amplitudes and latencies for wave I were also measured at 8, 16, and 32 kHz from 90 dB for all animals. A wave I amplitude was determined by measuring the voltage difference between the highest positive value (peak) and greatest negative value (trough) for the first ABR wave as previously described by Chen et al. [25]. A wave I latency was measured as the amount of time elapsed from the onset of the stimulus and the offset of the first wave (trough). We used 7–8 mice per group for ABR threshold, amplitude, and latency assessments. Following the ABR hearing measurements, tissues from the same mice were used to conduct histopathological analyses.

### Cochlear histology

#### Histological evaluation

Following the ABR hearing measurements, the animals were sacrificed by cervical dislocation and the temporal bone was excised from the head and divided into cochlear and vestibular parts [23]. The cochlea was then excised, immersed in a fixative containing 4% paraformaldehyde (Sigma-Aldrich) in phosphate buffered saline (PBS) solution for 1 d, decalcified in 10% EDTA for 1 week, and embedded in paraffin. The paraffin-embedded specimens were sliced along the mid-modiolar axis into 5 μm sections, mounted on silane-coated slides, stained with H&E, and observed under a light microscope (Leica). Rosenthal's canal was divided into three regions: apical, middle and basal and the three regions were used for evaluation of cochlear histology. We used 4–5 mice per group for histopathological assessment. In each mouse, we evaluated every third modiolar section obtained from one cochlea for a total of ten sections. Tissues from the same animals were used for cochleograms, spiral ganglion neuron (SGN) counting, and stria vascularis thickness measurement.

#### Cochleogram

The number of inner hair cells (IHC), first-row outer hair cells (OHC1), second-row outer hair cells (OHC2), and third-row outer hair cells (OHC3) were counted over 0.24 mm intervals along the entire length of the cochlea under the microscope at 400X magnification as previously described [26, 27]. The counting results were then entered into a custom computer program designed to compute a cochleogram that shows the number of missing IHC and OHC1-3 as a function of percentage distance from the apex of the cochlea. Frequency-place map for mouse cochlea was shown on the abscissa in the figures as previously described [28, 29].

#### Spiral ganglion neuron counting

SGN were counted in the apical, middle, and basal regions of the cochlear sections using a 40x objective as previously described [23]. Type I and type II neurons were not differentiated, and cells were identified by the presence of a nucleus. The corresponding area of the Rosenthal canal was measured in digital photomicrographs of each canal profile. The perimeter of the canal was traced with a cursor using ImageJ software (National Institutes of Health). The computer then calculated the area within the outline. The SGN survival was calculated as the number of SGN per mm^2^. Six to nine sections of the apical, middle, and basal turns were evaluated in one cochlea per mouse. We used 4–5 mice per group for SGN counting.

#### Stria vascularis thickness measurements

Stria vascularis thickness was measured in 40X images of H&E-stained mouse cochlear tissues. In the ImageJ software (National Institutes of Health), the measurement was made by using a cursor to draw a line from the margin of the stria to the junction of the basal cells with the spiral ligament half-way between the attachment of Reissner’s membrane and the spiral prominence [30]. Measurements were made at the basal, middle and apical regions of the cochlea for each mouse, and averages of each region were calculated for each mouse. Six to nine sections of the apical, middle and basal turns were evaluated in one cochlea per mouse. We used 4–5 mice per group for stria vascularis thickness measurements.

### Isolation of cytosol, nuclei, and mitochondria

Labyrinth tissues including bony shell, cochlear lateral wall, cochlear basilar membrane, cochlear modiolus, utricle, saccule, and three semicircular canals were homogenized using a tissue grinder (Wheaton dounce tissue grinder, Fisher Scientific) containing 1 ml of Tris buffer (10 mM Tris, 1 mM EDTA, 320 mM sucrose, pH 7.4) on ice and then centrifuged at 720 g (3000 rpm) for 5 min at 4°C to get a nuclear fraction (pellet). The supernatant was centrifuged at 12000 g for 10 min at 4°C to get a mitochondrial fraction (pellet) and a cytosolic fraction (supernatant). The pellets (nuclear fraction and mitochondrial fraction) were re-suspended vigorously with 200 μl or 100 μl of 1% NP-40 buffer (50 mM Tris, 250 mM NaCl, 1% NP40, pH 7.4) and after 30 min of incubation on ice, were centrifuged at 12000 g for 10 min at 4°C. Lamin B1, VDAC, or GAPDH was used as a nuclear, mitochondrial, or cytosolic marker (loading control) respectively.

### Western blotting

Fifty μg of total protein were fractionated by 10% of SDS-PAGE and transferred to nitrocellulose membranes (Bio-rad). Membranes were incubated with the primary antibody followed by the horseradish peroxidase-linked secondary antibody. A chemiluminescent detection reagent (ECL Prime, GE Healthcare Life Sciences) was used to visualize proteins. The band intensity was quantified using the ImageJ software (National Institutes of Health) and the levels of each protein were normalized by loading controls. Primary antibodies used were as follows: Gsr (rabbit polyclonal, 1:1000 dilution, Abcam), Lamin B1 (rabbit polyclonal, 1:2000 dilution, Abcam), VDAC (rabbit polyclonal, 1:1000 dilution, Cell Signaling), GAPDH (rabbit polyclonal, 1:5000 dilution, Abcam). Secondary antibodies used were as follows: rabbit (1:5000 dilution, GE Healthcare Life Sciences) secondary antibodies.

### Measurement of oxidative damage markers

#### Oxidative DNA damage marker

The level of the oxidative DNA damage marker 8-oxoguanine was analyzed using the Oxyselect Oxidative DNA Damage ELISA kit (Cell Biolabs) according to the manufacturer’s instructions. In brief, the 96 well plate was coated with 8-OHdG conjugate (1 μg/ml) and DNA extracted from inner ears was converted to single-stranded DNA at 95°C for 5 min and was cooled down on ice. DNA samples were digested to nucleosides by incubating with 5–20 units of nuclease P1 (Sigma-Aldrich) for 2 h at 37°C in a final concentration of 20 mM Sodium acetate, pH 5.2, followed by treatment of 5–10 units of alkaline phosphatase (Sigma-Aldrich) for 1 h at 37°C in a final concentration of 100 mM Tris, pH 7.5. The reaction mixture is centrifuged for 5 min at 6000 g and the supernatant is used for the 8-OHdG ELISA assay. Fifty μl of samples or 8-OHdG standards are added to the wells of the 8-OHdG conjugate-coated plate and incubated for 10 min at room temperature on an orbital shaker. Fifty μl of the diluted anti-8-OHdG antibody was added to each well and incubated for 1 h at room temperature on an orbital shaker. After washing with 1x washing buffer three times, 100 μl of the diluted secondary antibody-enzyme conjugate was added to all wells and incubated at room temperature for 1 h on an orbital shaker. After washing with 1x washing buffer three times, 100 μl of substrate solution was added to each well and incubated for 10 min at room temperature. The reaction was stopped by adding 100 μl of stop solution into each well. The Absorbance was read at 450 nm in a spectrometer (Bio-Tek).

#### Oxidative protein damage marker

The level of the oxidative protein damage marker protein carbonyl was analyzed using the Oxyblot Protein Oxidation Detection kit (EMD Millipore) according to the manufacturer’s instructions. In brief, 8 μg of cytosolic lysate were denatured by adding the same volume of 12% SDS for a final concentration of 6% SDS and were derivatized by adding 2 volumes of 1x DNPH Solution to the tubes and incubated at room temperature for 15 min. One and half volume of Neutralization Solution was added to tubes to stop reaction. 2-mercaptoethanol (1–1.5μL; 5% v/v) was added to tubes to achieve a final concentration of 0.74 M solution to reduce samples. Samples were loaded into a polyacrylamide gel (4–20%) (Bio-rad) and separated at 100 V for 90 min. Proteins on the gel were transferred to the nitrocellulose membrane (Bio-rad). The membrane was incubated with the blocking buffer (4% skim milk in PBS) for 1 hour. The membrane was incubated with the primary antibody (1:150, diluted in the blocking buffer) overnight at 4°C. The membrane was washed with PBS-T containing 0.05% (v/v) tween-20 (Sigma-Aldrich) in PBS for 10 min three times. The membrane was incubated with the secondary antibody (1:300, diluted in the blocking buffer) for 1 hour at room temperature. The membrane was washed with PBS-T for 10 min three times. The membrane was developed with Amersham ECL Prime (GE healthcare). The intensity of bands was quantified using ImageJ software (National Institutes of Health).

### Measurement of antioxidant activities

#### Glutathione peroxidase activity

Glutathione peroxidase activity was measured using the Glutathione Peroxidase Assay kit (Sigma-Aldrich) according to the manufacturer’s instructions. In brief, 80 μl of cytosolic lysate was added to a well in the 96 well plate and then 120 μl of mixture containing 10 μl of NADPH reagent (5 mM NADPH, 42 mM reduced glutathione, and 10 U/ml glutathione reductase), 50 μl of assay buffer (50 mM Tris-HCl, pH 8.0, 0.5 mM EDTA), and 60 μl of 30 mM *tert*-butyl hydroperoxide was added to the well. The Absorbance was read at 340 nm every 10 s for 1 min in a spectrometer (Bio-Tek) to calculate the activity. All samples were run in duplicate.

#### Glutathione reductase activity

Glutathione reductase activity was measured using the Glutathione Reductase Assay kit (Sigma-Aldrich) according to the manufacturer’s instructions. In brief, 20 μl of cytosolic lysate was added to a well in the 96 well plate and then 180 μl of mixture containing 50 μl of 1 mM GSSG, 20 μl of assay buffer, 50 μl of 0.75 mM DTNB, and 60 μl of 0.1 mM NADPH was added to the well. The Absorbance was read at 405 nm every 10 s for 2 min in a spectrometer (Bio-Tek) to calculate the activity. All samples were run in duplicate.

#### Glutamate-cysteine ligase activity

Glutamate-cysteine ligase activity was measured as previously described [31]. In brief, 30 μl of cytosolic lysate was mixed with 30 μl of GCL-reaction cocktail containing 400 mM Tris-HCl, 40 mM ATP, 40 mM L-glutamic acid, 2 mM EDTA, 20 mM sodium borate, 2 mM serine, and 40 mM MgCl_2_. After incubation at 37°C for 5 min, 30 μl of 30 mM cysteine solution in TES/SB buffer was added and the mixtures were incubated at 37°C for 13 min. The enzymatic reaction in the mixtures was stopped by precipitation of proteins with 200 mM 5-sulfosalicylic acid (SSA). After placing the mixtures on ice for 20 min, the mixtures were centrifuged at 2,000 g at 4°C for 10 min. Twenty μl of each supernatant that contained the γ-GC product was added to a 96 well plate. Twenty ul of γ-GC standards containing 5 μl of GCL reaction cocktail, 5 μl of 200 mM SSA, 5 μl of H_2_O, and 5 μl of γ-GC standard solution (0, 20, 40, 60. 80, 120, or 140 μM γ-GC in TES/SB buffer) was added to appropriate wells. TES/SB buffer (w/v = 1/4) consists of 20 mM Tris-HCl, 1 mM EDTA, 250 mM sucrose, 20 mM sodium borate and 2 mM serine, and 180 μl of 2,3-naphthalenedicarboxyaldehyde (NDA) was added into each well. After incubation in the dark at room temperature for 30 min, the formation of NDA-γ-GC was measured (472 nm excitation/ 528 nm emission) using a fluorescent plate reader (Bio-Tek). The production of γ-GC in each sample was calculated with a standard curve. The values are expressed in mM/min/mg protein. All samples were run in duplicate.

#### Catalase activity

Catalase activity was measured using the Catalase Assay kit (Sigma-Aldrich) according to the manufacturer’s instructions. In brief, 25 μl of cytosolic lysate (5~10 μg protein/μl) was mixed with 50 μl of 1x assay buffer and 25 μl of 200 mM H_2_O_2_ solution and incubated for 2 min at room temperature. The reaction was stopped by adding a stop solution (15 mM sodium azide in water). Then, 10 μl out of the 100 μl reaction mixture was mixed with 990 μl of the color reagent (150 mM potassium phosphate buffer, pH 7.0, containing 0.25 mM 4-aminoantipyrine and 2 mM 3,5-dichloro-2-hydroxybenzensulfonic acid) in a new tube by inversion. After 15 min of incubation for color development, the absorbance was measured at 520 nm in a spectrometer (Bio-Tek). Activity (μmoles/min/mg protein or U/mg protein) was calculated using the equation “Δμmoles (H_2_O_2_) = A520 (Blank)—A520 (Sample).” All samples were run in duplicate.

#### Superoxide dismutase activity

Superoxide dismutase (SOD) activity was measured as previously described [32]. In brief, a separating gel was made by mixing 8.48 ml of double distilled water, 7.28 ml of Acyl-Bis 30% (w/v), 2.25 ml of Tris separating buffer, pH 8.8 (1.5 M Tris and 8 mM disodium EDTA) and 9 μl of TEMED, and 68 μl of ammonium persulfate and was polymerized for 20–30 min at room temperature. A stacking gel mixed with 1 ml of Acyl-Bis 30% (w/v), 1.6 ml of Tris stackging buffer, pH 6.8 (0.5 M Tris and 8 mM disodium EDTA), 3.2 ml of sucrose 40% (w/v), 800 μl of riboflavin-5-phosphate 0.004% (w/v), and 4 μl of TEMED was added to the surface of the running gel and was polymerized under a fluorescent light for 30 min. The gel was pre-electrophoresed for 1 h, at 40 mA at 4°C in a pre-electrophoresis buffer, pH 8.3 (187 mM Tris and 1 mM disodium EDTA) to remove residual APS, TEMED and incomplete polymerization products, which may inactivate native proteins. Samples (50 μg of cytosolic lysate) were prepared by mixing with the same volume of sample loading buffer containing 10 ml of Tris stacking buffer, 10 ml of glycerol, and 200 μl of bromophenol blue solution 5% (w/v). Samples were run in the pre-electrophoresis buffer for 3 h at 40 mA at 4°C and then were run in the electrophoresis buffer, pH 8.3 containing 50 mM Tris, 300 mM glycine, and 1.8 mM disodium EDTA for 3 h at 40 mA at 4°C. The gel was stained with 40 ml of SOD native gel stain containing 2.43 mM nitro blue tetrazolium chloride, 170 ul of 28 mM TEMED, 8 μl of 0.14 M riboflavin-5’-phosphate (dissolved in 50 mM phosphate buffer, pH 7.8) in a plastic container for 20 min at room temperature, shaking in the dark or covered by foil. After incubation, the gels were gently washed with double distilled water (ddH_2_O) twice and sufficient ddH_2_O were added to cover the gel. The gels were placed under a fluorescent light until the gels turn blue/purple and clear bands appear gradually. Achromatic bands indicate the presence of SOD. The bands on the gels were imaged on a flatbed scanner and were quantified in the ImageJ software (National Institutes of Health). All reagents used in this assay were purchased from the Sigma-Aldrich company. All samples were run in duplicate.

#### Thioredoxin reductase activity

Thioredoxin reductase activity was measured using the Thioredoxin Reductase Assay kit (Sigma-Aldrich) according to the manufacturer’s instructions. In brief, 10 μl of cytosolic lysate was added to wells in the 96 well plate and then 190 μl of mixture containing 180 μl of working buffer (100 mM potassium phosphate, 10 mM EDTA, and 0.24 mM NADPH), 6 μl of 100 mM DTNB, and 4 μl of either 1 x assay buffer (100 mM potassium phosphate, pH 7.0, 10 mM EDTA) or thioredoxin reductase inhibitor was added to the wells. The Absorbance was read at 412 nm every 10 s for 2 min in a spectrometer (Bio-Tek) to calculate the activity. All samples were run in duplicate.

#### Thioredoxin activity

Thioredoxin activity was measured using the Thioredoxin Activity Fluorescent Assay kit (Cayman chemical) according to the manufacturer’s instructions. In brief, 30 μl of cytosolic lysate was added to wells in the 96 well plate and then 45 μl of mixture containing 35 μl of working buffer (0.1 mg/ml bovine serum albumin, 50 mM Tirs-HCl and 1 mM EDTA, pH 7.5) and 10 μl of 0.74 U thioredoxin reductase was added to all wells. Five μl of β-NADPH was added to each well and the plate was covered with a lid and incubated at 37°C for 30 min. 20 μl of the fluorescent substrate was added to each well and the plate was incubated for 5 min at room temperature. The active thioredoxin was measured (520 nm excitation/ 545 nm emission) using a fluorescent plate reader (Bio-Tek). The level of active thioredoxin in each sample was calculated with a standard curve. The values are expressed in ng/μg protein. All samples were run in duplicate.

### Measurement of Total GSH and GSSG

Labyrinth tissues were homogenized using a tissue grinder (Wheaton dounce tissue grinder, Fisher Scientific) containing 1 ml of lysis buffer (10 mM Tris, 20 mM EDTA, 320 mM sucrose, pH 7.4) on ice and then centrifuged at 12000 g for 10 min at 4°C to get a cytosolic lysate (supernatant). One hundred μl of the cytosolic lysate was used for the measurements of cytosolic glutathione contents. Total glutathione (GSH + GSSG) and GSSG levels were determined by the method of Rahman et al [33]. The rates of 5’-thio-2-nitrobenzoic acid (TNB) formation were calculated, and the total glutathione (tGSH) and GSSG concentrations in the samples were determined by using linear regression to calculate the values obtained from the standard curve. The GSH concentration was determined by subtracting the GSSG concentration from the tGSH concentration. All samples were run in duplicate. All reagents used in this assay were purchased from the Sigma-Aldrich.

### Measurement of NADPH

NADPH levels were determined by the method of Zerez et al. [34]. Briefly, 200 μl of the cytosolic lysate was mixed with 180 μl of a nicotinamide solution (10 mM nicotinamide, 20 mM NaHCO_3_, and 100 mM Na_2_CO_3_) and freeze-thawed three times to extract NADP^+^ and NADPH. To destroy NADP^+^ in the samples, 90 μl of the mixture was incubated in a heating block for 30 min at 60°C. Twenty-five μl of each unheated and heated sample was mixed with 225 μl of a reaction mixture (100 mM Tris, 5 mM EDTA, 0.5 μM thiazolyl blue tetrazolium bromide, 2 μM phenazine ethosulfate, and 1.3 units glucose-6-phosphate dehydrogenase, pH 8.0) and incubated for 5 min at 37°C. The reaction mixture was then transferred to each well of a 96-well plate and 1 ml of 1 mM glucose-6-phosphate was added in each well to start the reaction. The absorbance was read at 570 nm every 10 sec for 3 min in a spectrometer (Bio-Tek). The reaction rates were calculated and NADPH levels were determined as the ratio of NADPH (heated sample) to the total of NADP^+^ and NADPH (unheated sample). All reagents used in this assay were purchased from the Sigma-Aldrich. All samples were run in duplicate.

### Glutathione S-transferase activity

Glutathione S-transferase activity was measured using the Glutathione S-Transferase Assay kit (Sigma-Aldrich) according to the manufacturer’s instructions. In brief, 20 μl of cytosolic lysate was added to a well in the 96 well plate and then 180 μl of mixture containing 176.4 μl of Dulbecco’s phosphate buffered saline (DPBS), 1.8 μl of 200 mM L-Glutathione reduced, and 1.8 μl of 100 mM 1-Chloro-2,4-dinitorbenzene (CDNB) was added to the well. The Absorbance was read at 340 nm every minute for 6 min in a spectrometer (Bio-Tek) to calculate the activity. All samples were run in duplicate.

### Cytochrome c oxidase activity

Cytochrome *c* oxidase activity was measured using the Cytochrome *c* Oxidase Assay kit (Sigma-Aldrich) according to the manufacturer’s instructions. In brief, 10 μl of cytosolic lysate was added to wells in the 96 well plate and then 210 μl of mixture containing 190 μl of assay buffer (50 mM Tris-HCl, pH 7.0 and 600 mM KCl), 10 μl of enzyme dilution buffer (20 mM Tris-HCl, pH 7.0 and 500 mM sucrose), and 10 μl of 0.22 mM ferrocytochrome c substrate solution was added to the wells. The Absorbance was read at 550 nm every 5 s for 1 min in a spectrometer (Bio-Tek) to calculate the activity. All samples were run in duplicate.

### Statistical analysis

The distributions of the three genotypes *Gsr*^+/+^, *Gsr*^+/-^, and *Gsr*^-/-^ for male, female and combined male and female were compared separately with the expected distribution calculated by Mendel's Laws of Inheritance (0.25:0.5:0.25). The Chi-squared test was used to do the comparison as previously described [35]. The chi-squared value was calculated based on the formula of $x^{2}=\sum_{k=1}^{n}\frac{{\left(O_{k}-E_{k}\right)}^{2}}{E_{k}}$, where *O*_*k*_ is the observed count of the cell k and *E*_*k*_ is the expected count of the same cell. The significance threshold was set to 0.05. One-way ANOVA with post-Tukey multiple comparison tests (GraphPad Prism 4.03) was used to analyze the Gsr protein levels in subcellular fractions. Two-way ANOVA with Bonferroni’s *post hoc* test (GraphPad Prism 4.03) was used to analyze the ABR thresholds, losses of SGN, thickness of stria vascularis, and cochleogram. Student’s t-test was used to analyze the body weight, antioxidant enzyme activities, oxidative damage markers, and GSH/GSSG.

## Results

### Localization of GSR in mouse cochlea

**Fig 2:**
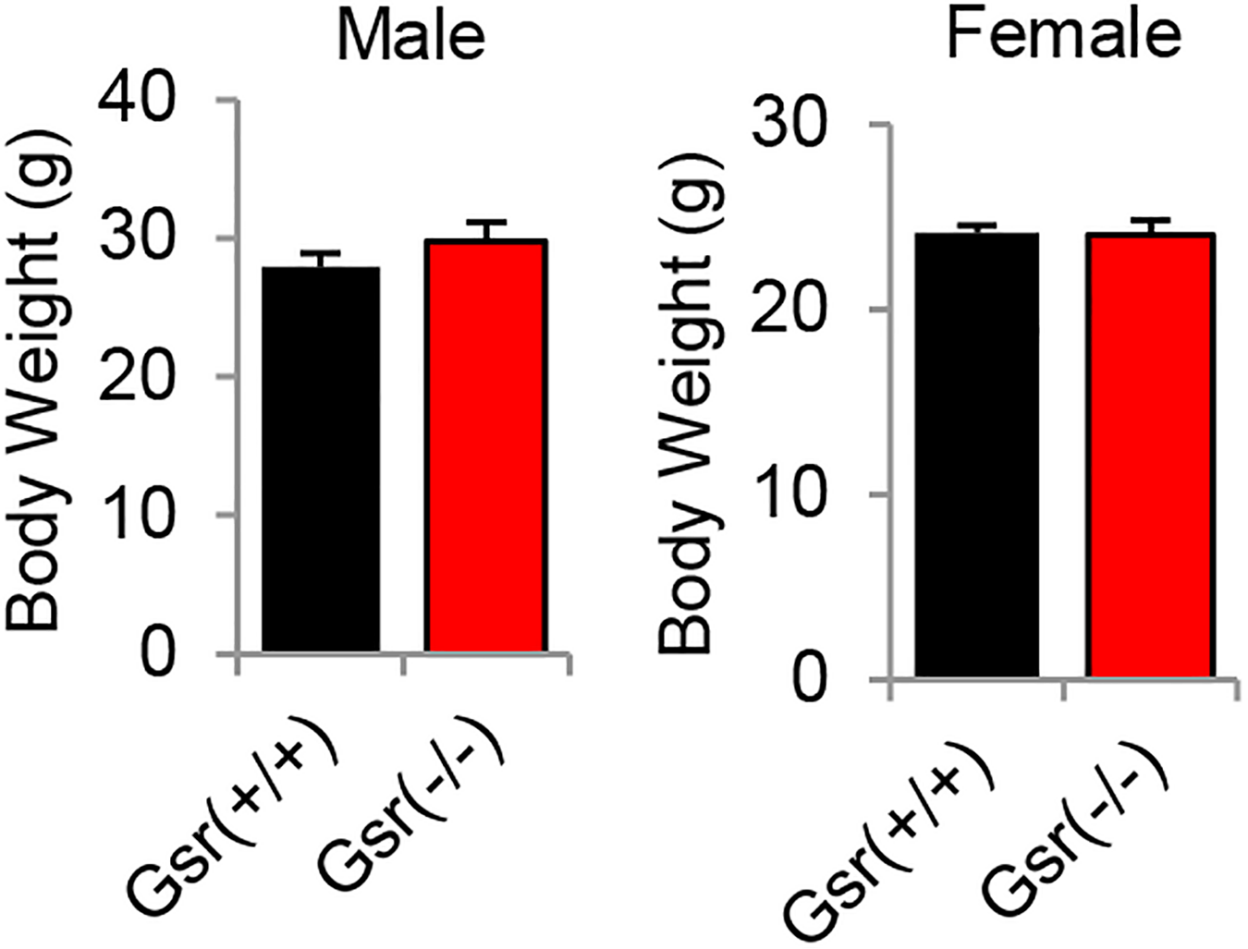
Body weight of young *Gsr*^+/+^ and *Gsr*^-/-^ mice. Body weights of male and female *Gsr*^+/+^ and *Gsr*^-/-^ mice were measured at 3–5 months of age (N = 10–11). Data are shown as means ±SEM.

To investigate the roles of GSR in the antioxidant defense in mouse cochlea, *Gsr*^+/-^ mice were backcrossed for 6 generations onto the CBA/CaJ mouse strain, a normal-hearing strain that does not carry the recessive early-onset hearing loss-susceptibility allele (*Cdh23*^753A^) [22, 36]. We identified *Gsr*^+/+^, *Gsr*^+/-^, and *Gsr*^-/-^ mice by PCR genotyping (Fig 1A) and then sequenced the *Cdh23* gene in the DNA obtained from tails of young *Gsr*^+/+^, *Gsr*^+/-^, and *Gsr*^-/-^ mice. We confirmed that all *Gsr*^+/+^, *Gsr*^+/-^, and *Gsr*^-/-^ mice had the same wild-type genotype (*Cdh23*^753G/753G^) (Fig 1B). Mice lacking the *Gsr* gene on the CBA/CaJ background appeared phenotypically normal, and no significant changes were observed in body weight between *Gsr*^+/+^ and *Gsr*^-/-^ males or females (Fig 2). To further investigate the fertility and viability of *Gsr*^+/-^ and *Gsr*^-/-^ males or females, the genotype distributions of *Gsr*^+/+^, *Gsr*^+/-^, and *Gsr*^-/-^ for male, female and combined male and female were compared separately with the expected distribution calculated by Mendel's Laws of Inheritance (0.25:0.5:0.25) using the Chi-squared test. The offspring from *Gsr*^+/-^ mating were born at the expected Mendelian ratios, indicating that *Gsr*^-/-^ are viable on the CBA/CaJ background (Table 1). These observations are consistent with the phenotypic data of *Gsr* homozygous knockout mice on the 129SvEv^Brd^;C57BL/6J background reported by Lexicon Genetics (http://www.informatics.jax.org/external/ko/lexicon/454.html).

**Table 1:** Fertility of *Gsr*^-/-^ mice.

Sex		*Gsr*^+/+^	*Gsr*^+/-^	*Gsr*^-/-^	Total
Combined	Expected	59.25	118.5	59.25	237
	Observed	71	102	64	237
Male	Expected	31.75	63.5	31.75	127
	Observed	34	58	35	127
Female	Expected	27.5	55	27.5	110
	Observed	37	44	29	110

The observed numbers for male and female mice of the three genotypes shown in the table were obtained from heterozygous intercrosses. The Expected numbers were calculated based on the Mendelian inheritance. The Chi-Square tests results are: Combined, *X*(2) = 5.0084, p = 0.0817; Male, *X*(2) = 0.9685, p = 0.6162; Female, *X*(2) = 5.5636, p = 0.0619.

**Fig 3:**
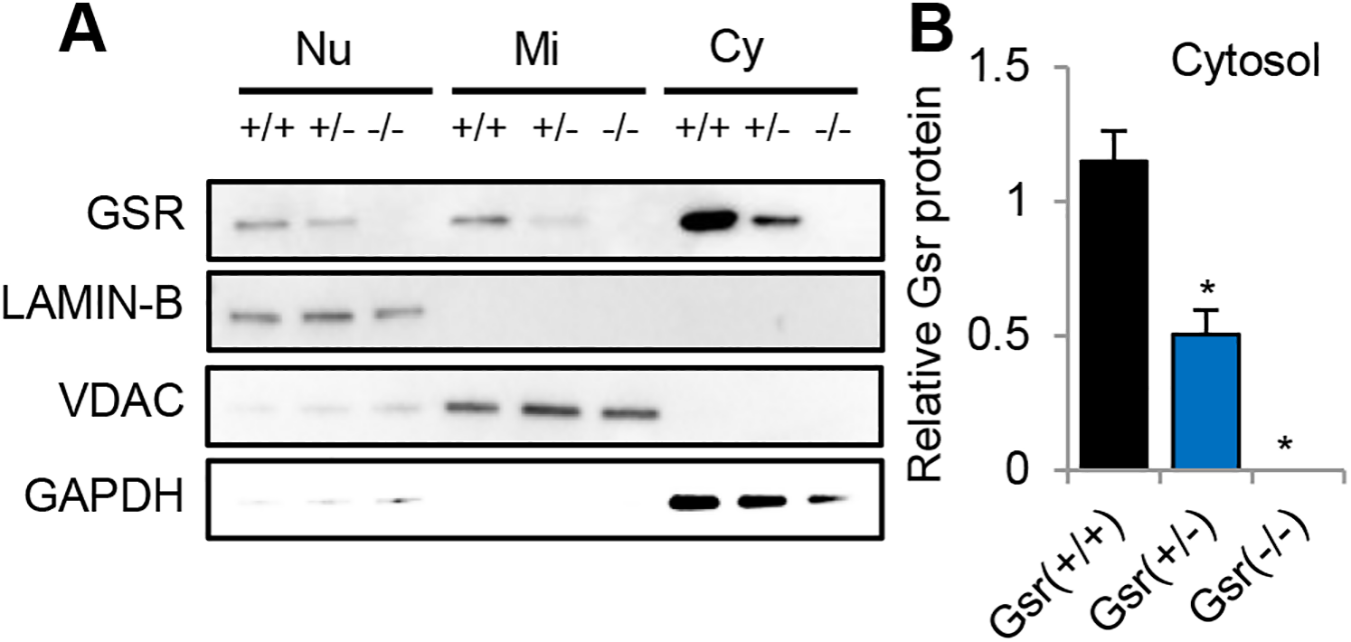
Localization of GSR protein. (A) Western blotting analysis of GSR protein levels in the inner ear from young *Gsr*^+/+^, *Gsr*^+/-^, and *Gsr*^-/-^ mice. (B) Quantification of GSR proteins in the cytosol in the inner ear tissues from 3–5 months old *Gsr*^+/+^, *Gsr*^+/-^, and *Gsr*^-/-^ mice. Lamin B1, VDAC, and GAPDH were used as nuclear, mitochondrial, and cytosolic markers (loading control) respectively. Nu = nuclear, Mi = mitochondria, Cy = cytosol. Data are shown as means ±SEM. (N = 3). *p<0.05 vs. *Gsr*^+/+^.

To investigate the subcellular localization of GSR protein in the inner ears, we measured GSR protein levels in the cytosol, nuclei, and mitochondria of the inner ear tissues from 3–5 months old WT and *Gsr*^-/-^ mice by western blotting. *Gsr*^+/-^ mice displayed a 45% decrease in GSR protein levels, while *Gsr*^-/-^ mice displayed no detectable level of GSR in the inner ears (Fig 3A and 3B). In WT mice, GSR protein was detected in all the subcellular compartments; however, the most intense GSR protein band was observed in the cytosol (Fig 3A). GSH is synthesized in the cytosol, from glutamate, cysteine, and glycine by cytosolic glutamate-cysteine ligase (GCL) and glutathione synthetase (GS), and most (85–90%) of the cellular glutathione is present in the cytosol [2–4]. Therefore, our western blotting results suggest that GSR is mostly present in the cytosol of mouse inner ears.

#### *Gsr* deficiency does not affect hearing function in mice

**Fig 4:**
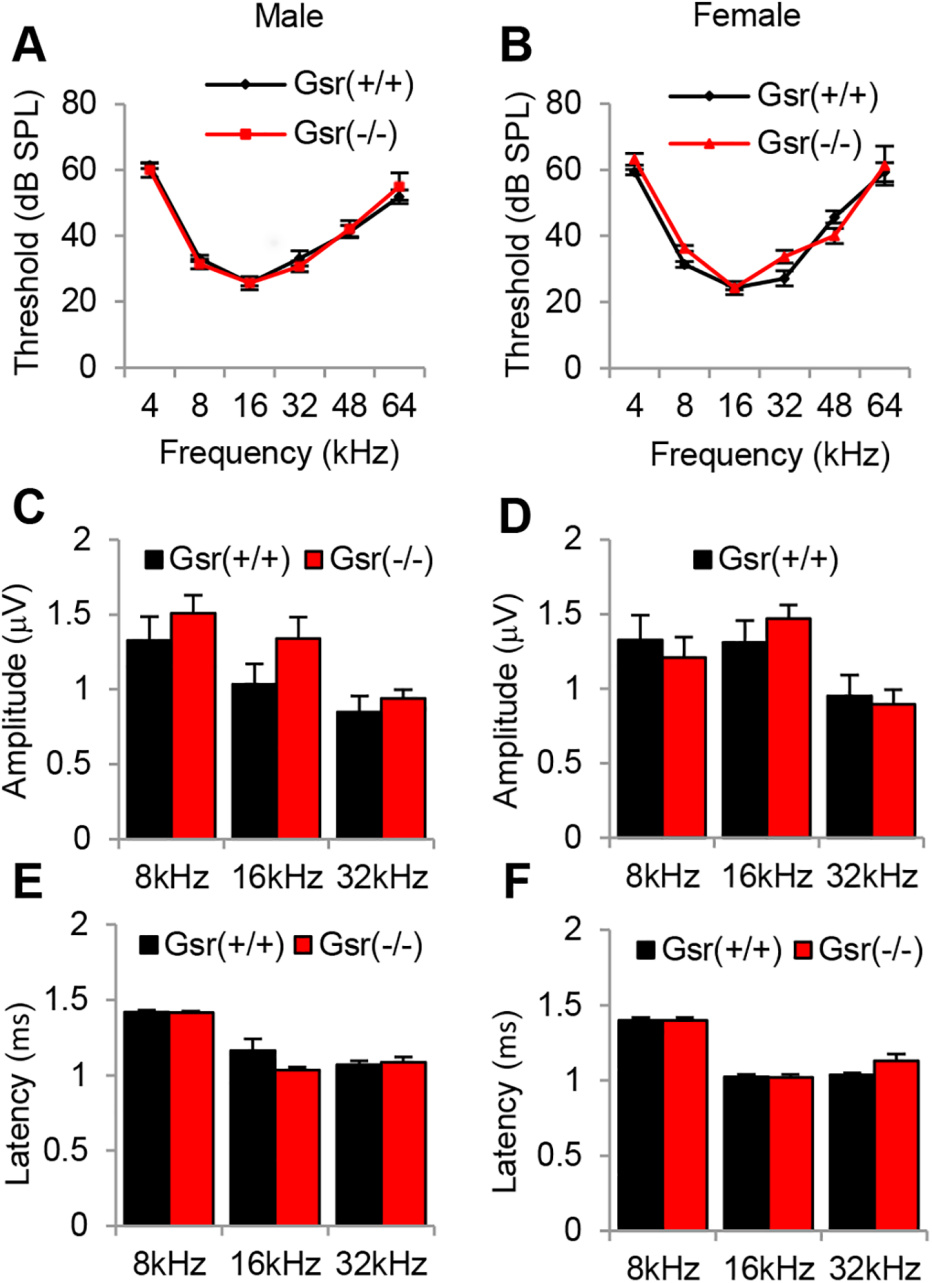
Assessment of ABR hearing thresholds, amplitudes and latencies in young *Gsr*^+/+^, *Gsr*^+/-^, and *Gsr*^-/-^ mice. ABR hearing thresholds were measured at 4, 8, 16, 32, 48 and 64 kHz in 3–5 month-old *Gsr*^+/+^ and *Gsr*^-/-^ males (A) and females (B) (N = 7–8). ABR amplitudes and latencies of wave I at 90 dB at 8, 16, and 32 kHz from 3–5 month-old *Gsr*^+/+^ and *Gsr*^-/-^ mice in males (C, E) and females (D, F) (N = 7–8). Data are shown as means ±SEM.

If ROS play a causative role in sensorineural hearing loss, then a defect in GSR may result in increased oxidative damage in the cochlea and affect hearing function at a younger age. To test this hypothesis, we measured ABR (auditory brainstem response) hearing thresholds, wave I amplitudes and wave I latencies in *Gsr*^+/+^ and *Gsr*^-/-^ males and females at 3–5 months of age. There were no differences in the ABR thresholds at 4, 8, 16, 32, 48 or 64 kHz between *Gsr*^+/+^ and *Gsr*^-/-^ males or females (Fig 4A and 4B). In agreement with the ABR threshold results, there were no differences in the wave I amplitudes or latencies at 8, 16 or 32 kHz between *Gsr*^+/+^ and *Gsr*^-/-^ males or females (Fig 4C–4F). Together, these physiological analysis results show that *Gsr* deficiency does not affect hearing function in young mice that are on the CBA/CaJ background.

#### *Gsr* deficiency does not increase oxidative damage in mouse cochlea

**Fig 5:**
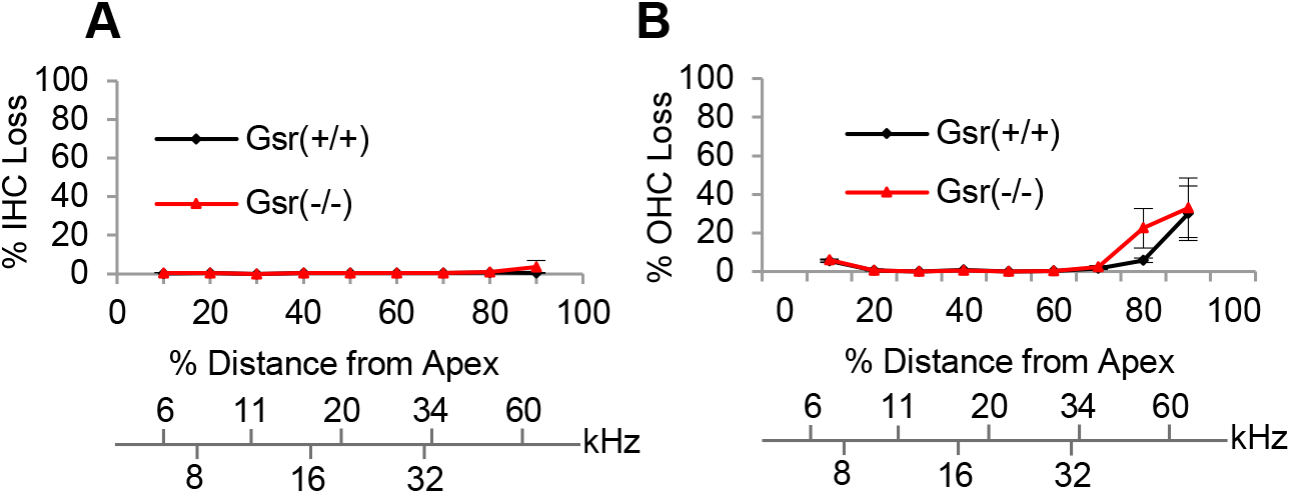
Cochleograms of young *Gsr*^+/+^ and *Gsr*^-/-^ mice. Cochleograms were recorded and averaged in the cochlear tissues of 3–5 month-old *Gsr*^+/+^ and *Gsr*^-/-^ males (N = 4–5). The graphs show the quantification of cell loss in the inner hair cells (IHC) (A) and outer hair cells (OHC) (B). Data are shown as means ±SEM.

**Fig 6:**
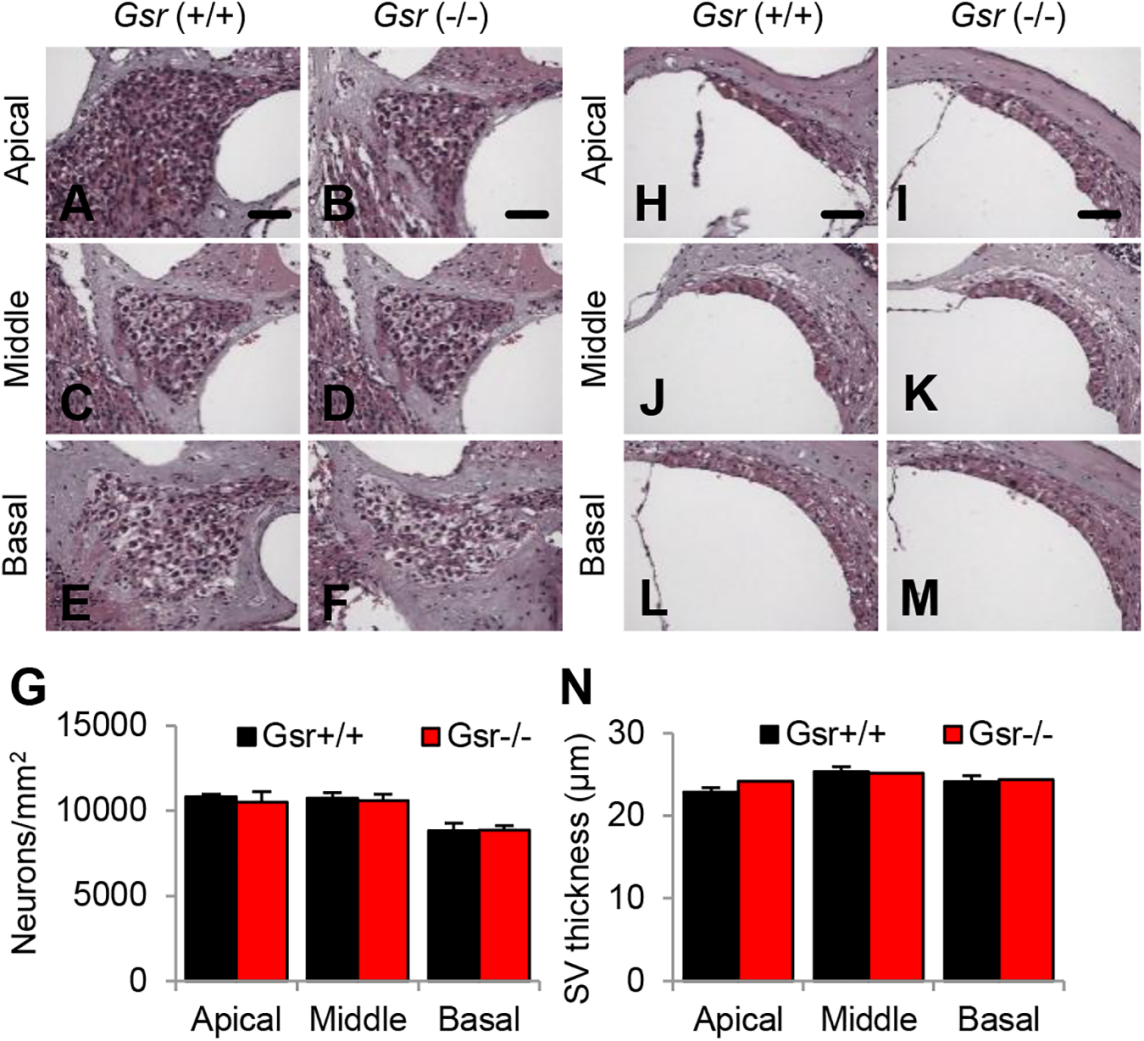
Histological analysis of cochlear spiral ganglion neuron density and stria vascularis thickness in young *Gsr*^+/+^ and *Gsr*^-/-^ mice. (A-G) The densities of spiral ganglion neurons (SGN) in the apical, middle, and basal regions of cochlear tissues from 3–5 month-old male *Gsr*^+/+^ and *Gsr*^-/-^ mice (N = 4–5) were counted and quantified (G). (H-N) The thickness of stria vascularis (SV) in the apical, middle, and basal region of cochlear tissues from 3–5 month-old *Gsr*^+/+^ and *Gsr*^-/-^ males (N = 4–5) was measured (N). Data are shown as means ±SEM. Scale bar = 25 μm.

The major sites of cochlear pathology typically include sensory hair cells, spiral ganglion neurons (SGNs) and stria vascularis (SV) [37, 38]. First, to investigate whether *Gsr* deficiency results in increased oxidative damage in cochlear hair cells, mean cochleograms were prepared from 3–5 month-old *Gsr*^+/+^ and *Gsr*^-/-^ male mice. Because there were no sex differences in hearing function between *Gsr*^+/+^ or *Gsr*^-/-^, all the histological analyses were conducted in male mice. There were no or little inner hair cell (IHC) loss or outer hair cell (OHC) loss in the apical, middle or basal regions of the cochlea in both *Gsr*^+/+^ and *Gsr*^-/-^ mice, and there were no differences in the numbers of IHCs or OHCs between WT and *Gsr*^-/-^ mice (Fig 5A and 5B). Next, we counted the numbers of SGNs in the apical, middle, and basal regions of the cochlea from young *Gsr*^+/+^ and *Gsr*^-/-^ mice. There were no or minimal SGN loss in the apical, middle or basal regions of the cochlea in both *Gsr*^+/+^ and *Gsr*^-/-^ mice, and there were no differences in the densities of SGNs between WT and *Gsr*^-/-^ mice (Fig 6A–6G). Atrophy of the stria vascularis in the cochlea is one of the most prominent features of age-related hearing loss in humans [37–39]. To investigate whether *Gsr* deficiency results in SV degeneration in the cochlea, we measured the thickness of SV in the apical, middle, and basal regions of the cochlea from 3–5 month old *Gsr*^+/+^ and *Gsr*^-/-^ mice. In agreement with the hair cell and SGN counting results, there were no differences in the thickness of SV in the apical, middle or basal regions of the cochlea between *Gsr*^+/+^ and *Gsr*^-/-^ mice (Fig 6H–6N). Together, these histological and physiological analysis results indicate that GSR is not required for cochlear development in mice.

**Fig 7:**
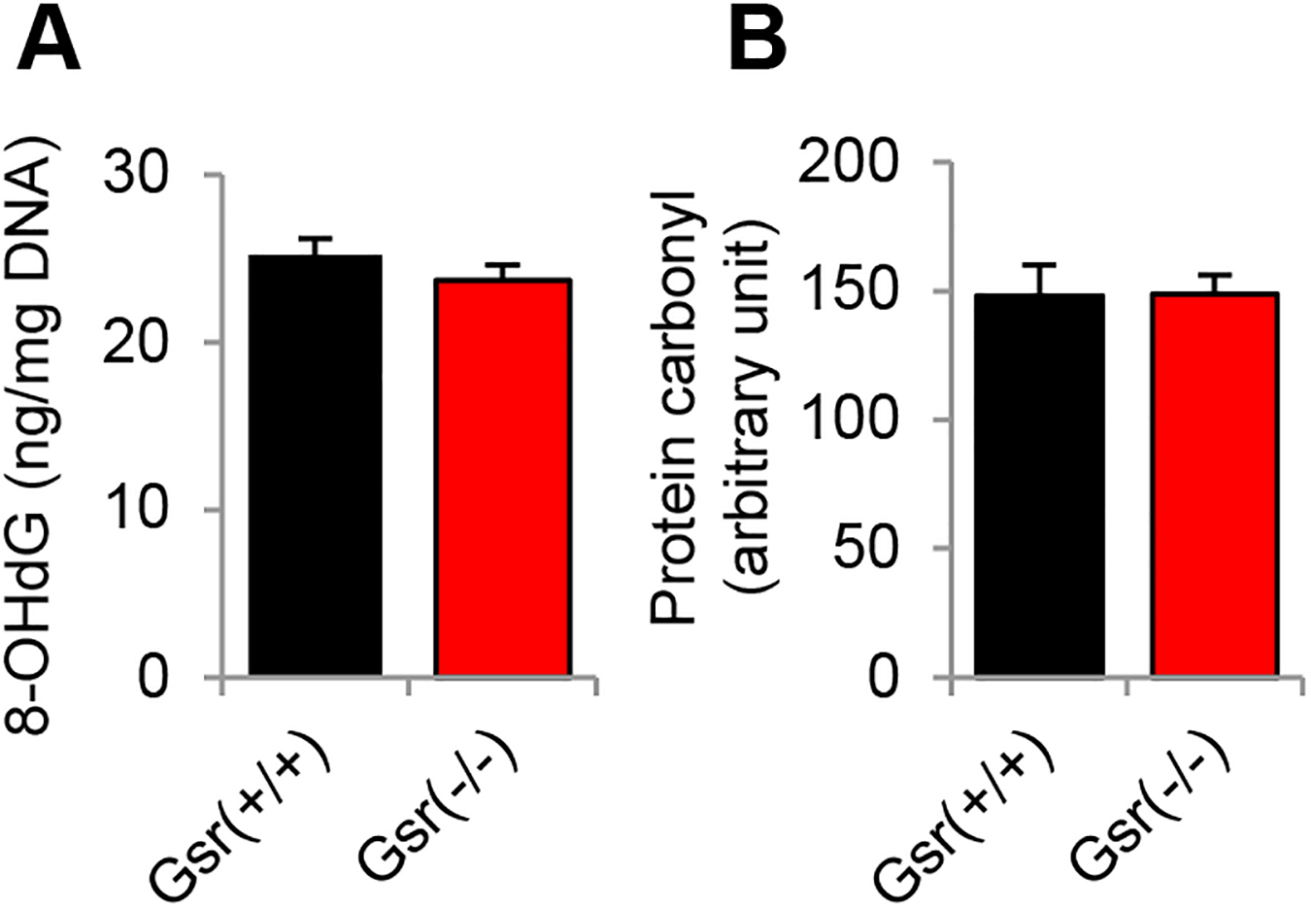
Assessment of oxidative DNA and protein damage in the inner ear tissues of young *Gsr*^+/+^ and *Gsr*^-/-^ mice. Levels of 8-oxoguanine (8-OHdG) as an oxidative DNA damage marker (A) and protein carbonyl (B) as an oxidative protein damage marker were measured in the inner ear tissues from 3–5 month-old *Gsr*^+/+^ and *Gsr*^-/-^ males (N = 4–5). Data are shown as means ±SEM.

If GSR plays an essential role in protecting cochlear cells against ROS, then a defect in GSR may result in increased oxidative damage in the cochlea. To test this hypothesis, we measured levels of the oxidative DNA damage marker 8-oxoguanine in the inner ears from young *Gsr*^+/+^ and *Gsr*^-/-^ mice. There were no differences in the levels of 8-oxoguanine in the inner ears between *Gsr*^+/+^ and *Gsr*^-/-^ mice (Fig 7A). We also measured levels of protein carbonyl, a marker of oxidative protein damage, in the inner ears from young *Gsr*^+/+^ and *Gsr*^-/-^ mice. Because GSR protein was predominantly detected in the cytosol in the inner ears of WT mice (Fig 3), protein carbonyl levels were measured in the cytosol of the cochlea. There were no differences in the levels of protein carbonyl in the inner ears between *Gsr*^+/+^ and *Gsr*^-/-^ mice (Fig 7B). Taken together, these histological and biochemical analysis results show that *Gsr* deficiency does not increase oxidative damage in the hair cells, spiral ganglion neurons or stria vascularis cells in the cochlea of mice.

#### *Gsr* deficiency decreases glutathione redox state, but not the activities of glutathione-related antioxidant enzymes in the cytosol of the cochlea

**Fig 8:**
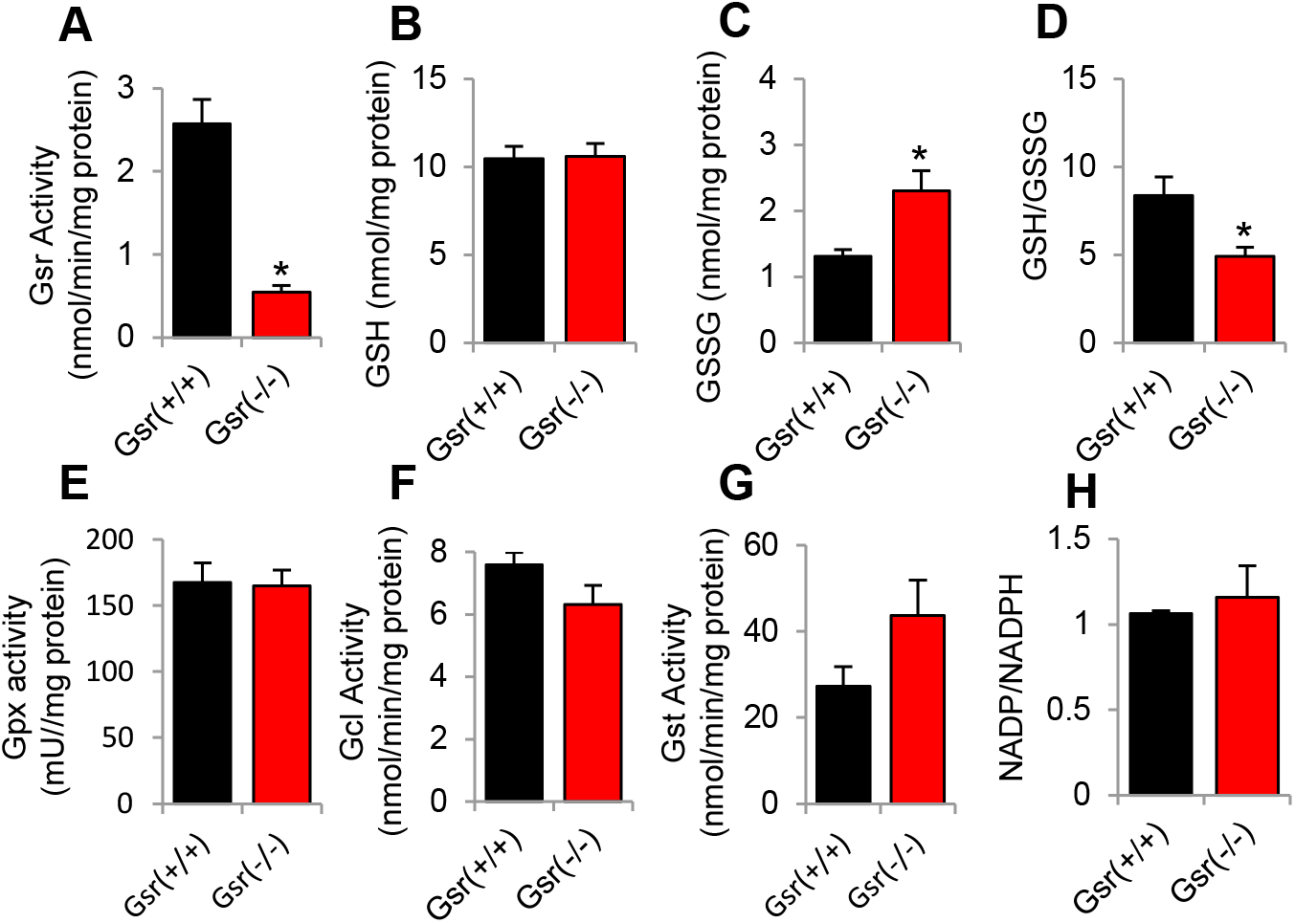
Biochemical analysis of cytosolic glutathione-related enzymatic activities and GSH/GSSG ratios in the inner ear tissues of young *Gsr*^+/+^ and *Gsr*^-/-^ mice. The activities of GSR (A), the levels of GSH (B), GSSG (C), and GSH/GSSG (D), the activities of GPX (E), GCL (F), and GST (G), and the levels of NADPH (H) were measured in the cytosol of the inner ear tissues from 3–5 month-old *Gsr*^+/+^ and *Gsr*^-/-^ males (N = 4–5). Data are means ±SEM. *p<0.05 vs. *Gsr*^+/+^. GSR = glutathione reductase, GPX = glutathione peroxidase, GCL = glutamate-cysteine ligase, GST = glutathione transferase.

The glutathione antioxidant defense system is thought to be the major line of defense against ROS in the cochlea [11–17]. To investigate if *Gsr* deficiency affects the activities of glutathione-related enzymes in mouse cochlea, we measured activities of GSR, GPX, GCL, and GST, and the levels of GSSG and total glutathione in the inner ear tissues from *Gsr*^+/+^ and *Gsr*^-/-^ male mice at 3–5 months of age. Because GSR protein was predominantly detected in the cytosol in the inner ears of WT mice (Fig 3), all the measurements were performed in the cytosol of the inner ears. *Gsr*^-/-^ mice displayed an 80% decrease in GSR activity in the inner ears compared to WT mice (Fig 8A). This is consistent with the previous report that GSR activity was diminished by 98% in the red blood cells, 92–97% in the liver and kidney, and 86% in the brain of *Gsr* hypomorphic mice [7]. *Gsr*^-/-^ mice also displayed a 40% decrease in the GSH/GSSG ratios in the inner ears compared to WT mice (Fig 8B–8D). However, there were no significant differences in the activities of GPX between WT and *Gsr*^-/-^ mice (Fig 8E). Furthermore, there were no differences in the activities of GCL, the first and rate-limiting enzyme in glutathione synthesis [40], between *Gsr*^+/+^ and *Gsr*^-/-^ mice (Fig 8F). There were also no differences in the activities of glutathione transferase in the inner ears between *Gsr*^+/+^ and *Gsr*^-/-^ mice (Fig 8G). Glutathione reductase requires NADPH, an essential cofactor and reductant for regeneration of GSH from GSSG [3, 7, 8]. To investigate whether *Gsr* deficiency affects NADPH levels, we measured NADPH levels in the inner ear tissues from young *Gsr*^+/+^ and *Gsr*^-/-^ mice. There were no differences in the levels of NADPH between WT and *Gsr*^-/-^ mice (Fig 8H). Together, these biochemical analysis results show that *Gsr* deficiency significantly decreases GSR activity and GSH/GSSG ratios; however, the resulting decline in the glutathione redox status does not affect the activities of GPX, GCL, GST nor NADPH levels in the cytosol of the inner ears.

**Fig 9:**
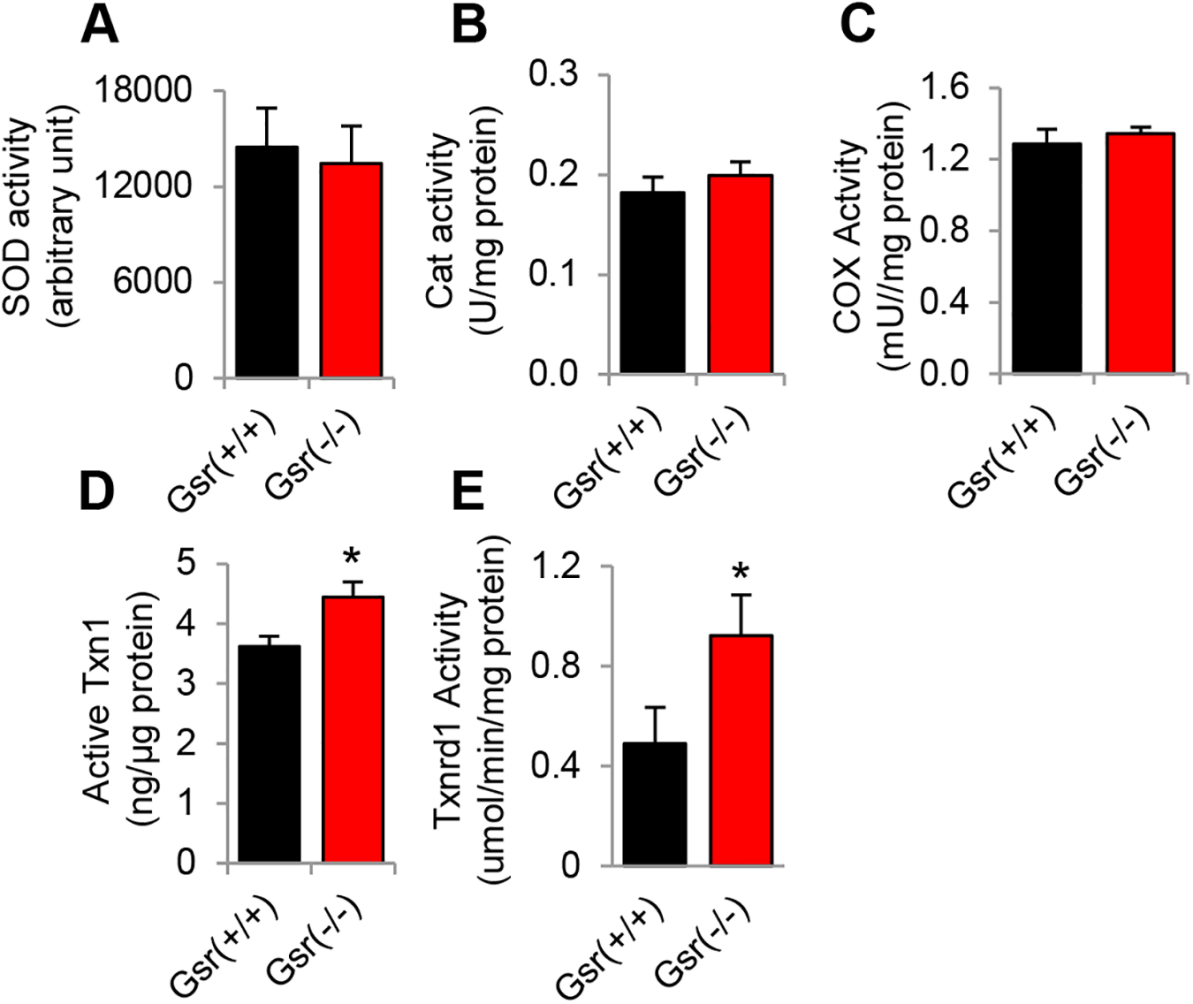
Biochemical analysis of cytosolic antioxidant enzymatic activities in the inner ear tissues of young *Gsr*^+/+^ and *Gsr*^-/-^ mice. The activities of cytosolic SOD (A), catalase (B), COX (C), TXN1 (D), and TXNRD1 (E) were measured in the inner ear tissues from 3–5 month-old *Gsr*^+/+^ and *Gsr*^-/-^ males (N = 4–5). Data are shown as means ±SEM. *p<0.05 vs. *Gsr*^+/+^.

To investigate whether *Gsr* deficiency affects the activities of other antioxidant enzymes in mouse inner ears, we measured the activities of superoxide dismutase (SOD) that decomposes superoxide into oxygen and hydrogen peroxide [1], catalase (Cat) that decomposes hydrogen peroxide into water [1], and cytochrome *c* oxidase (COX), a marker of mitochondrial membrane integrity [41], in the cytosol of the inner ear tissues from *Gsr*^+/+^ and *Gsr*^-/-^ male mice at 3–5 months of age. There were no differences in the activities of SOD, catalase or COX between young *Gsr*^+/+^ and *Gsr*^-/-^ mice (Fig 9A–9C), suggesting that *Gsr* deficiency does not affect the total antioxidant defense in mouse inner ear. The question then becomes what antioxidant enzyme or antioxidant system can support GSSG reduction in the absence of GSR. Previous reports have shown that the thioredoxin-thioredoxin reductase system can support GSSG reduction as a functional backup for GSR in parasites, plants, yeast, flies, and mice [42–46]. Therefore, we reasoned that the cytosolic thioredoxin-thioredoxin reductase system may support GSSG reduction in the absence of GSR in mouse inner ear. To test this hypothesis, we measured the activities of cytosolic thioredoxin and thioredoxin reductase in the inner ear from young *Gsr*^+/+^ and *Gsr*^-/-^ mice. We found that *Gsr*^-/-^ mice displayed increased activities of thioredoxin and thioredoxin reductase compared to WT mice (Fig 9D and 9E), suggesting that the thioredoxin-thioredoxin reductase system can support GSSG reduction in the absence of GSR in mouse inner ear.

## Discussion

We have demonstrated that GSR is not required for cochlear development and function in young mice. We found that *Gsr* homozygous knockout mice are viable and appear normal. These observations are consistent with the previous report that *Gsr* hypomorphic mice are viable and appear healthy, [7] and the phenotypic data of *Gsr* homozygous knockout mice on the 129SvEv^Brd^;C57BL/6J background characterized by Lexicon Genetics (http://www.informatics.jax.org/external/ko/lexicon/454.html). In the current study, young *Gsr* homozygous knockout mice displayed an 80% decrease in GSR activity in the cytosol of the inner ears (Fig 8A), despite the fact that *Gsr*^-/-^ mice displayed no detectable level of GSR in the inner ear (Fig 3A and 3B). We speculate that the GSR activity assay used in the current study could have measured the activities of residual NADPH-dependent thioredoxin reductase in the sample. Histological analysis also revealed that no pathological abnormalities were observed in the cochlea of *Gsr*^-/-^ mice (Figs 5 and 6). In agreement with the histological observations, there were no significant differences in the ABR hearing threshold, wave I amplitude or wave I latency between WT and young *Gsr*^-/-^ males or females (Fig 4). Therefore, these results provide concrete evidence that GSR is not required for cochlear development and function in young mice. In light of the widely-accepted view that the GSH/GSSG redox couple is a determinant of the cellular redox state and plays important roles in antioxidant defense and cellular survival [4, 6], the finding that knockout of *Gsr* does not cause any cochlear histological abnormalities is rather surprising. There is also a large body of evidence indicating that GSH is essential for development and survival: although glutathione synthetase (*Gss*) heterozygous knockout mice are viable and appear normal, *Gss* homozygous knockout mice die before embryonic day 7.5 [47]. In the *Gss* heterozygous knockout mice, GSS activity was diminished by 50% in the liver, kidney, brain, and spleen. A complete knockout of the *Gclc* (glutamate cysteine ligase catalytic subunit) gene that encodes a catalytic subunit of GCL, also results in embryonic lethality in mice, whereas *Gclc* heterozygous knockout mice are viable and fertile [48]. GCL activity was diminished by 45%, while GSH levels were diminished by 20% in the liver of *Gclc* heterozygous knockout mice. In agreement with these reports, *Gpx1* homozygous knockout mice exhibit 16 dB higher ABR thresholds at 28.3 kHz under normal physiological conditions, although these KO mice show no developmental, neurological or reproductive abnormalities [18]. *Sod1* deficiency also leads to minor (0–7 dB) threshold elevations at 5 and 10 kHz under normal physiological conditions, although *Sod1* homozygous knockout mice develop normally [49]. Furthermore, the glutathione redox state declines in the liver, kidney, heart, testis, eye and brain of mice during aging [6], while old long-living dwarf mice display significantly higher activities of GCL and GST in the kidney when compared to age-matched wild-type mice [50]. In humans, *GSS* deficiency is associated with hemolytic anemia, severe brain malformations, respiratory failure and early death [51]. Approximately 25% of *GSS* deficient patients died during the neonatal period. Therefore, we speculate that depletion of GSH and a decrease in the GSH redox state below a certain threshold will likely cause cochlear histological abnormalities and associated hearing loss during development.

The GSH/GSSG redox couple is thought to be an intracellular determinant of the antioxidant capacity within the cochlea [11–17]. This is in part because the abundance of GSH is 3–4 orders of magnitude higher than the other reductants, including NADPH, NADH, cysteine, GRX_red_ (reduced glutaredoxin) and TXN_red_ (reduced thioredoxin), and GSH has a lower standard redox potential. In support of this view, depletion of GSH by the GSH synthesis inhibitor BSO results in greater temporary and permanent threshold shifts and greater OHC loss in the basal region of the cochlea of guinea pigs after noise exposure [15], suggesting that depletion of GSH in the cochlea can potentiate the susceptibility of the cochlea to noise trauma. Another early study has shown that GSH levels were significantly increased in the lateral wall of the cochlea in guinea pigs following noise exposure [13], while sound conditioning that reduces NIHL, increases the activities of GSR and GCL in the organ of Corti and stria vascularis of the cochlea of chinchilla following acute noise exposure [16]. Importantly, Meniere’s disease patients, whose symptoms include hearing loss, exhibit a significant decrease in the GSH/GSSG ratios in the plasma and lymphocyte [11]. However, in the current study, we found that there were no differences in the activities GPX, GCL or GST in the cytosol of the inner ears between young *Gsr*^+/+^ and *Gsr*^-/-^ mice, despite the fact that *Gsr*^-/-^ mice displayed a 40% decrease in the GSH/GSSG ratios in the cytosol of the inner ears (Fig 8A and 8D). There were also no differences in the activities of catalase or SOD in the cytosol of the inner ears between *Gsr*^+/+^ and *Gsr*^-/-^ mice. Furthermore, there were no differences in the levels of oxidative DNA or protein damage marker in the inner ears between *Gsr*^+/+^ and *Gsr*^-/-^ mice (Fig 7). These results are consistent with the histological observations that no histological abnormalities were found in the cochlea of *Gsr*^-/-^ mice. Therefore, it is likely that a decline in the GSH/GSSG redox state that does not reach a certain threshold, does not affect antioxidant defense function in the cochlea. Our findings also suggest that in the absence of GSR, the GSH/GSSG redox couple may not be the primary intracellular determinant of the antioxidant capacity within the cochlea.

The finding that a significant decline in the GSH/GSSG ratio due to the *Gsr* deficiency does not affect the activities of glutathione-related enzymes strongly suggests that there is another reductant system that can contribute to GSSG reduction in the mouse peripheral auditory system. Previous reports have shown that the thioredoxin system can support GSSG reduction as a functional backup for GSR in multiple species: The thioredoxin system is one of the major antioxidant defense systems against ROS through its disulfide reductase activity in cells [52]. The thioredoxin system is composed of thioredoxin, thioredoxin reductase (TXNRD), peroxiredoxin (PRX) and NADPH. NADPH-dependent TXNRD catalyzes the reduction of oxidized TXN (TXN_oxi_) to regenerate reduced TXN (TXN_red_). Subsequently, TXN_red_ catalyzes the reduction of oxidized PRX (PRX_oxi_) to regenerate reduced PRX (PRX_red_) which then decompose hydrogen peroxide into water. In 2000, Kanzok et al [42] showed that GSSG reduction can be supported at a high rate by the thioredoxin/thioredoxin reductase system in glutathione reductase-deficient cells in malaria parasites. In the following year, the same group demonstrated that the thioredoxin/thioredoxin reductase system supports GSSG reduction in the absence of glutathione reductase in Drosophila melanogaster [45]. In plants, GSSG is reduced to GSH by two glutathione reductase isoforms, cytosolic GSR1 and GSR2 that are localized in the mitochondria and chloroplast [43]. Marty et al found that the NADPH-dependent thioredoxin system supports GSSG reduction as a functional backup for cytosolic GSR1 in Arabidopsis. In yeasts, Tan et al [44] have shown that the cytosolic thioredoxin/thioredoxin reductase system, but not the glutaredoxin system, can support GSSG reduction in cells lacking the *GSR* gene. Furthermore, deletion of cytosolic *TXN1* or cytosolic *TXN2* in the *GSR*-deficient cells resulted in increased accumulation of GSSG. Consistent with those reports, *Gsr* hypomorphic mice display a 50% increase in cytosolic TXN1 protein levels and a 75% increase in mitochondrial TXN2 protein levels in the liver, compared to control mice [46]. In the current study, we have demonstrated that knockout of *Gsr* results in increased activities of cytosolic thioredoxin and thioredoxin reductase in the inner ear. Therefore, these previous reports and our data suggest that in the absence of GSR, the thioredoxin/thioredoxin reductase system likely supports GSSG reduction as a functional backup for GSR in mouse cochlea under normal physiological conditions. In summary, under normal physiological conditions, GSR is not essential for the maintenance of antioxidant defenses in mouse cochlea.
